# Applying Principles of Biomechanics of the Spine to Martial Arts: A Review on Balance of Stances in Goju-Ryu Karate-Do

**DOI:** 10.3390/jfmk11010011

**Published:** 2025-12-26

**Authors:** Michael Fiechter, Tobias Pötzel, Marc E. Pfeifer

**Affiliations:** 1 Swiss Paraplegic Center, Spine Surgery, 6207 Nottwil, Switzerland; 2 Swiss Paraplegic Research, 6207 Nottwil, Switzerland; 3 Swiss Goju-Ryu Karate-Do Renmei (SGRKR), 6330 Cham, Switzerland; marc.pfeifer@hevs.ch; 4 Institute of Life Sciences, School of Engineering, University of Applied Sciences and Arts Western Switzerland (HES-SO Valais-Wallis), 1950 Sion, Switzerland

**Keywords:** karate, Goju-Ryu, balance, alignment, spine, stance

## Abstract

Balance is referred to as a state of equilibrium where forces of agonist and antagonistic muscles are equal. This is particularly relevant in the practice of Goju-Ryu Karate-Do, a martial art style with combinations of hard and soft techniques. Particularly, karate stances not only depend on technical aspects but also on the ability to achieve a centered posture. In this narrative review, we aim to integrate the existing knowledge about alignment parameters of the spine to various stances in Goju-Ryu Karate-Do to improve biomechanical understanding, allow technical modifications, and consequently enhance positive training experience. Balance is constantly challenged during the interplay of accelerated movements and subsequent controlled postures (Japanese: “Kamae”). This requires continuous neuromuscular coordination to maintain the body’s equilibrium. In particular, the body’s center of gravity, which is located around the second sacral vertebra in a standing position, needs to be kept within Dubousset’s “efficiency cone” for low energy consumption and minimal fatigue. This state is primarily maintained by aligning the spine, the pelvis, and the lower extremities, which is a result of complex biomechanical interactions of various spinopelvic parameters. Applying these concepts of Dubousset to stances in Goju-Ryu Karate-Do helps to explain why during the aging process or natural degeneration, technical modifications are needed to guarantee an optimal training experience in senior or disabled practitioners of Karate-Do. Biomechanical parameters of the spinopelvic axis are crucial in mastering the art of Goju-Ryu Karate-Do. Only with a balanced stance and an optimally situated center of gravity, a block or attack may be successful and movement strategies effective. However, technical modifications of stances must be considered in aged or disabled karateka to allow a sustained training experience.

## 1. Background

Goju-Ryu Karate-Do, originating from Okinawa (Japan) and originally developed by Chojun Miyagi during the early 20th century, is a martial art style that combines both hard (Japanese: “Go”) and soft (Japanese: “Ju”) techniques as a harmonious approach to combat [1]. To effectively perform and master the entire range of techniques, a high skill level of coordination between fine and gross motor groups is required. Consequently, balanced stances are fundamental prerequisites in mastering Goju-Ryu Karate-Do. Only with an adequate posture is a balanced stance achievable, which in turn, enables an optimal training of body awareness and control (i.e., stability). Thus, balanced stances attune the body to the center of gravity (COG) which is situated within the pelvis and around sacral vertebra 2 in a standing position. The location of the COG predominantly decides whether blocks or attacks are effective. By extending the COG with a plumb line within the human body, it is comprehensible that both the spine and the pelvis play an important role in maintaining balance [2]. The fundamental concept of bipedal erect posture and balance was first described by Dubousset et al. in the 1970s, which led to the introduction of the cone of economy [3]. The cone of economy refers to imaginary boundaries around the human body, allowing humans to stand upright using minimal muscle activity and low energy consumption. Any deviation from this cone leads to a significant activity of the muscles groups to maintain the body’s equilibrium. This concept led to the understanding of the spinopelvic alignment and balance of both the spine and ultimately, the human body. For the assessment of the spinopelvic parameters, the spine is either classified by the Roussouly [4] and/or the SRS-Schwab [5] classification systems. However, the focus of these classifications resides primarily on the thoracolumbar spine. Regarding the global balance of the human body, the concept of the full body integrated (FBI) developed by Le Huec et al. [6] allows for the calculation of the orientation of the spine in relation to the hips and ankles, and thus, detects global malalignment and imbalance. The most frequently used parameters of spinopelvic alignment are the pelvic incidence (PI), the sacral slope (SS), the pelvic tilt (PT), the sagittal vertical axis (SVA), the thoracic kyphosis (TK), T1 pelvic angle (TPA), and the lumbar lordosis (LL) [7,8]. With these values, we not only establish an understanding of the spinopelvic alignment but also of the sagittal balance. However, age and natural degenerative processes significantly affect global balance. Amazingly, the ability to stand and walk remains preserved even at an advanced age and despite growing imbalance. This is explained by compensatory mechanisms which counteract any malalignment to maintain the balance of the human body [9,10]. The same mechanisms of compensation become equally important when it comes to the execution of stances in Goju-Ryu Karate-Do. Compensatory mechanisms allow technical modifications to maintain balanced stances and consequently, a sustained training experience even for aged or disabled practitioners. The objectives of this narrative review are to translate concepts of spinopelvic alignment and balance to martial arts with a focus on Goju-Ryu Karate-Do stances, to build an age- and spine-shape-dependent understanding relevant for practice, and to explore the limitations of age and deformity of the spine on training routines.

## 2. Impact of Biomechanics of the Spine to Stances in Goju-Ryu Karate-Do

### 2.1. Data Sources and Search Strategy

By the application of different combinations of keywords (“goju ryu” OR “karate” OR “martial art” OR “martial arts”) AND (“spinopelvic alignment” OR “sagittal balance” OR “spine”) in PubMed, we obtained 71 scientific articles focusing on the above-mentioned topics. Afterwards, we selected articles focusing on the key aspects of stances in Goju-Ryu Karate-Do with particular attention to those about the alignment and balance of the spine. Further, the references in the relevant articles were also screened and suitable articles were taken into consideration. Only scientific reports in the English language were further evaluated. Due to a lack of academic references in Goju-Ryu Karate-Do literature, a systematic review was not feasible and thus, no strict protocols were followed. Conclusively, this article must be perceived as a narrative review providing a summary with a synthesis of the existing literature in combination with the authors’ expertise and extensive experience.

### 2.2. The Dubousset’s Cone of Economy

A characteristic of the human’s locomotion is our bipedalism [11]. This transition from quadrupedal movement, as seen in apes, to bipedal gait in humans, has been a significant evolutionary step. When we adopt an upright posture, our skeleton, beginning at the feet and lower limbs, through the pelvis and spine, and finally to the skull, aligns harmoniously [12]. These components collectively form a ‘cone’ shape, with the body’s COG at the core of the structure. A healthy individual can maintain its balance within a small cone with minimal muscular effort, while a person with a locomotor system disorder requires a larger cone with maximum muscle effort to maintain balance. This principle, often referred to as the ‘cone of economy’ (introduced by Jean Felix Dubousset in the early 1970s), is now widely acknowledged [13]. The entire body’s neuro-musculoskeletal elements work together to achieve a balanced standing position, often referred to as the “chain of balance”. This “chain” supports the body and includes crucial body parts while the pelvis and the spine form its core. Only if the spinopelvic axis is aligned, a balanced posture can be achieved. As the spine ages and consequently, lumbar lordosis decreases, the thoracic spine flattens and the pelvis tilts backward while the knees start to bend (compensatory mechanism) to maintain an upright posture with a horizontal gaze. In individuals with advanced spinal deformities, the alignment of the lower limbs, particularly knee flexion, improves only after undergoing spinopelvic correction surgery [14,15]. This compensatory action is involuntarily carried out by the chain of balance.

### 2.3. Stances in Goju-Ryu Karate-Do

The importance of correct stances in martial arts, particularly in Goju-Ryu Karate-Do, should not be understated [16,17,18]. Stances serve as the foundation for all movements and techniques in martial arts. They provide stability, balance, and power. A correct stance allows a martial art practitioner to maintain balance, which is a prerequisite for both offensive and defensive techniques. It enables rapid and fluid transitions, enhancing the practitioner’s agility and speed. Moreover, a proper stance provides a solid base to generate power for effective techniques. In addition, stances play a strategic role in martial arts. The choice of stance relative to an opponent determines the effective range and type of techniques available as well as positional advantages. Therefore, mastering various stances expands the repertoire of fighting techniques and options for tactical moves especially in free fight (Japanese: “Jiyu Kumite”). Furthermore, practicing stances also contributes to the overall physical fitness and body awareness. It improves strength, particularly in the lower body and core muscles, enhances flexibility, and promotes better posture. The focus on body alignment in stances leads to improved proprioception. Goju-Ryu Karate-Do incorporates both circular and linear movements into its curriculum. To efficiently perform these techniques, different stances are necessary. A list of the basic stances is given in Table 1. Further, examples of the following stances are given in Supplementary Figures S1A–D and S2A,D–S4A,D.

Sanchin-, Shiko-, and Zenkutsu-dachi are the most frequently used stances in Goju-Ryu Karate-Do. Each of these stances are perfectly aligned with the spinopelvic axis located exactly at the center of the body and between the feet. The COG is distributed at different levels of height, lowest in Shiko-dachi and highest in Sanchin-dachi. Consequently, stability is maximized in Shiko-dachi and minimized in Sanchin-dachi. However, stability comes at a price, which is the reduction of agility. In Zenkutsu-dachi, the COG projects more towards the front leg, with approximately 66% of the weight placed on the front leg and the remainder on the rear leg. In Neko ashi-dachi, almost the entire weight and thus, the COG, is localized on the rear leg. All of these stances necessitate an upright posture and a balanced adjustment of the spine and pelvis, highlighting the intricate relationship between the COG, the posture, and the different stances in the practice of Goju-Ryu Karate-Do. Therefore, the mastery of stances is fundamental and forms the basis for the development of advanced techniques while contributing to physical fitness and body awareness. Hence, emphasis is placed on the technically correct execution of stances to achieve a balanced and stable posture.

### 2.4. Biomechanics of the Spine and Its Impact on Balance

**Figure 1:**
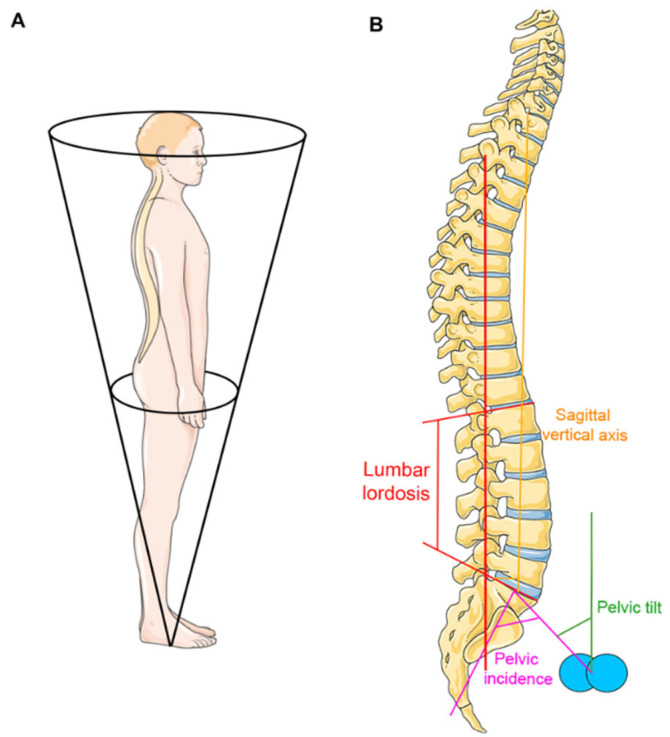
Sagittal alignment and balance of the spine. Maintaining balance in an upright stance requires the body to be situated within the cone of economy to keep balance using minimal muscle effort (**Panel A**). The most frequently used parameters of spinopelvic alignment are the pelvic incidence (PI), the pelvic tilt (PT), and the lumbar lordosis (LL), while one of the most established parameters to assess sagittal balance of the thoracolumbar spine is the sagittal vertical axis (SVA, (**Panel B**)). The blue spheres display the femoral heads. The figure was partly created using Servier Medical Art, provided by Servier, licensed under a Creative Commons Attribution 3.0 unported license.

Spinopelvic parameters play an important role in understanding the alignment and balance of the spine (Figure 1). These parameters offer an in-depth evaluation of the relationship between the spine and the pelvis, which is essential for maintaining an upright posture with minimal energy usage within the cone of economy [7,19]. The fundamental and most frequently used spinopelvic parameters include the PI, the SS, the PT, the SVA, the TK, the TPA, and the LL. The PI is a morphological parameter that remains unchanged irrespective of posture. It is the angle formed by the line perpendicular to the sacral plate at its midpoint and the line connecting this point to the axis of the femoral heads. The PI has a direct impact on the orientation of the pelvis, the LL, and overall sagittal balance. It also serves as a reference parameter to calculate the optimal LL. It classifies the spinopelvic alignment types to low vs. high PI (≤50° vs. >50°). The higher the PI, the greater the LL and the higher the infliction point (change from lordosis to kyphosis) of the lumbar spine. The PT is defined as the angle between the vertical and the line through the midpoint of the sacral plate to the axis of the femoral heads. The PT can increase when the knees are bent as the pelvis tilts backwards (retroversion) and reflects the magnitude of compensatory mechanisms in the case of deformity of the spine with sagittal imbalance. A physiological PT is usually located within 10–20°. The SS is the angle between the horizontal and the sacral plate. The SS can decrease when the knees are bent due to the posterior tilt (retroversion) of the pelvis. The SS is the primary determining parameter in the Roussouly classification [4] for sagittal alignment of the spine. The LL is the inward curvature of the lumbar spine, usually ranging from L1 to S1. The LL in younger adults is located from 40° to 60° while its ideal value is individual and matches the PI (approx. PI ± 10°). Further, the LL is a key parameter for global balance. The decline of the LL during age (the degeneration of intervertebral discs) leads to a flat back with sagittal imbalance once compensatory mechanisms fail. The SVA is a measure of thoracolumbar sagittal balance. It is the distance from the posterosuperior corner of S1 to the vertical line drawn from the center of C7. An increased SVA indicates a forward (anterior) shift of the body’s COG and thus, negatively impacts balance. Consequently, it is one of the most frequently used parameters to indicate sagittal imbalance (usually considered pathologic if greater than 5 cm). Finally, the TPA is the angle between the hips to the T1 line and the hips to the S1 endplate line. It provides a measure of the overall alignment of the spine in relation to the sacrum and is a novel and very promising parameter of the sagittal alignment of the spine. A value of <15° is usually considered physiologic with low disability while values >20° reflect sagittal malalignment with significant disability. Considering global balance, the FBI index measures the discrepancy between the current and ideal C7 plumb line position relative to the sacrum, considering compensatory angles from femoral obliqueness and pelvic tilt, to determine the optimal spinal alignment and global balance.

Both the aging process and degenerative pathologies may lead to spinopelvic malalignment, which is usually displayed by a loss of LL due to the degeneration of the lumbar intervertebral discs and consecutive loss of disc height. These mechanisms force the human body outside the cone of economy, which leads to imbalance and disability. However, compensatory mechanisms counteract this process by increasing the PT leading to a pelvic retroversion which maintains an upright posture. This goes along with bending the knees and a growing kyphosis of the cervical spine. Considering the SVA, knee flexion leads to a decrease in sagittal imbalance [20]. A decrease in the SVA shifts the COG back to a balanced state within the cone of economy. As for the TPA, there is limited information available on how knee flexion specifically affects this parameter. However, given that the pelvis tilts backwards, it is plausible to assume that the TPA, as a measure of the overall spinopelvic alignment in relation to the sacrum, is decreasing. The concept of spinopelvic alignment and balance is crucial, particularly in spine deformity surgery, as the restoration of the sagittal alignment and spinopelvic harmony is associated with favorable patient outcomes.

Turning now to karate stances, the most significant alteration to the upright standing position is bending the knees [21]. As mentioned above, knee flexion leads to significant changes in the spinopelvic parameters like pelvic retroversion and the alteration of the COG, which is usually lowered to increase stability. However, as the PI remains constant (morphological parameter that does not change with posture), bending the knees directly increases stress on the hips (by extension) and knees (by flexion). In healthy individuals, bending the knees results indirectly in a decrease in LL to keep a balanced stance. In persons with reduced LL (either due to age or degeneration), bending the knees is an involuntary reaction to counteract the lumbar hypolordosis and keep the spinopelvic axis balanced. In the case of a decreased range of motion (ROM) of these joints due to either osteoarthritis and shortened ligaments (i.e., due to poor physical condition) or spinal pathology, significant pain and disability can be provoked [22]. Further, if hip extension and knee flexion caused by spinal pathology is overlooked, the stress on these joints when performing a stance can further damage or even compromise the functioning of a knee and hip replacement [23,24].

In stances like Zenkutsu-dachi and Shiko-dachi, the maintenance of balance is fundamental, while compensatory mechanisms for sagittal imbalance of the spine prevent technically correct execution. The only way to still being able to perform these stances without harming the greater joints of the lower limbs is to raise the COG and accept a diminished ROM for both the greater joints and the spine. With this measure, the balance can be maintained despite spinopelvic malalignment. Nevertheless, the ability to absorb and execute strikes will be diminished. Thus, spinopelvic parameters can significantly impact the effectiveness of combat techniques due to the need for alterations of stances. Conclusively, a comprehensive understanding of spinopelvic alignment and its impact on karate stances can enhance the practice and mastery of karate, especially for persons with disability of the spine and greater joints as well as for aged practitioners.

### 2.5. Age-Dependent Impact on Balance

**Figure 2:**
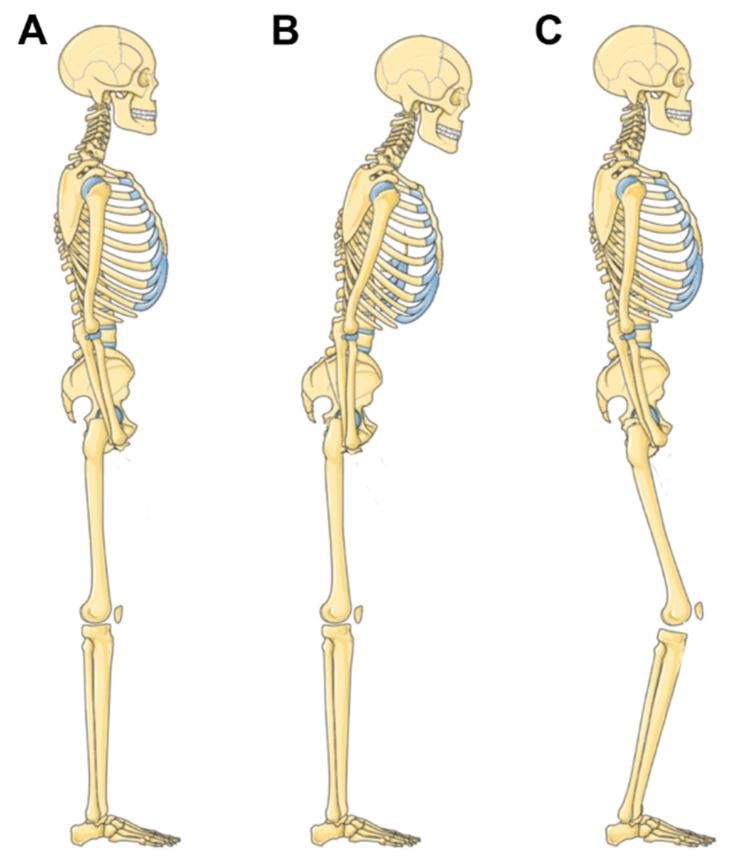
Sagittal imbalance and compensatory mechanisms. In healthy individuals, an upright posture requires minimal muscle activity due to spinopelvic alignment and balance (**Panel A**). In the case of aging or degenerative disease of the spine, the lumbar lordosis is decreased while spinopelvic malalignment develops with sagittal imbalance (**Panel B**). By compensatory mechanisms such as knee flexion, the pelvis is retroverted and the lumbar hypolordosis is compensated while the spinopelvic axis returns to a balanced state (**Panel C**). This figure was partly created using Servier Medical Art, provided by Servier, licensed under a Creative Commons Attribution 3.0 unported license.

Several spinopelvic parameters undergo age-dependent changes (Figure 2) [25,26]. The SVA gradually increases, shifting the head and upper body forward relative to the pelvis. This mainly results from a continuous degeneration of the intervertebral discs, which lose water and consequently decrease in height. As a result of this, the LL, including cervical lordosis, decreases and the PT increases which leads to the retroversion of the pelvis. Additionally, the TK increases, becoming increasingly rigid. These age-dependent and primarily degenerative processes have a critical impact on sagittal alignment (global kyphosis) and force the body gradually outside the cone of economy and as such, out of its balance. Further, the degeneration of the hip with reduced flexibility and elasticity leads to altered posture, which affects the spine by shifting the body weight anteriorly. This may lead to increased stress on the spine and surrounding anatomical structures. Notably, the body mass index (BMI) also significantly impacts spinopelvic alignment thresholds [27]. Integrating this gradual and age-dependent decompensation of the sagittal alignment of the spine into Karate-Do explains why at an advanced age, the ability to perform technically correct stances decreases and consequently, both movement execution efficiency and balance are negatively impacted. Age-dependent global kyphosis and rigidity of the spine together with reduced flexibility of the hips promote a progressive adult spine deformity, which is ultimately a natural development when growing older. As these deformities are mostly rigid, the body needs compensatory mechanisms to keep its core balance and thus allow for correct stances [28,29]. Interestingly, both the toddler and the aged human have a positive SVA where compensatory mechanisms are in action to keep global balance. In young or middle-aged healthy adults, the spine is usually perfectly balanced without the necessity for compensation. Any countermeasures against sagittal imbalance aim to preserve bipedal ability and are usually conducted unconsciously (Table 2).

Notably, only a few of these compensatory measures can be actively trained and adapted. These are mainly knee flexion and ankle/hip extension. Particularly, physical therapy can help to strengthen muscles, increase ROM, and improve posture. At a certain stage of deformity, lumbar back pain develops and if conservative measures such as physical therapy, pain killers, and interventional measures (i.e., infiltrations) fail, surgical correction might remain the only option to improve this disabling condition caused by imbalance [14,15,30]. However, decision-making towards the most suitable therapeutic strategy is complex and frequently requires sophisticated surgical approaches [31,32,33]. This starts with simple one-level fusion and ends up in complex and multi-level reconstructive surgery [34,35,36]. Despite the potential complications, surgical outcomes following adult spine deformity surgery are favorable regarding patient outcomes, with a significant improvement in activity and the quality of daily life [37], particularly in patients with preoperative physical therapy [38,39]. Thus, continuous training and optimization of the ROM of the greater joints such as ankles, knees, and hips are critical to maintain independence as long as possible. Both knee flexion and hip/ankle extension are connected to each other. They are basic elements for the ability to stand and walk but also to perform karate stances. Further, they contribute to stability, power generation, and effective execution of techniques. An overview of the functional purposes of knee flexion and hip/ankle extension in karate stances is provided in Table 3.

Maintaining a high ROM of the greater joints of the lower extremities with constant flexion of the knees in karate stances is essential for both correct technical performance and injury prevention. Whether a practitioner is a beginner or an expert, the focus should be on mastering the balance between stability and agility, also referred to as dynamics. However, for aged or disabled practitioners the following general training adaptations should be taken into consideration, as suggested in Table 4 [40].

### 2.6. Technical Modifications of Karate Stances in Aged or Disabled Individuals

In aged or disabled practitioners, technical modifications should be considered for injury prevention. While the effectiveness of strikes and blocks might be reduced, the probability to remain an active Karateka also at higher age significantly increases. Particularly, Zenkutsu-, Shiko-, and Neko ashi-dachi are technically demanding stances where slight modifications in the case of disability still allow a balanced stance. Heiko- and Sanchin-dachi are not further discussed here as the correct technical performance of these stances is independent from age and most disabilities. In Zenkutsu-dachi, aged practitioners may have a reduced ROM and strength in their lower extremities. Thus, the length of the stance can be shortened, and the front knee might be bent less deeply. This lessens stress on the joints and muscles while still maintaining the effectiveness and balance of the stances. However, this raises the COG which makes the stance perceived more stable, but it reduces the potential power which could be generated. In Shiko-dachi, the stance requires a wide leg position with deep knee bends, which can be challenging for individuals with knee or hip issues. Reducing the bending of the knees as well as the wideness of the stance reduces the stress on the greater joints. However, by raising the depth of the stance and thus the COG, balance is maintained but at the price of reduced stability. In Neko ashi-dachi, the stance demands balancing primarily on the posterior leg, which can be difficult for older individuals with a reduced ROM of the greater joints and balance issues. The capability of ankle and hip extension limits the optimal performance of this stance. Keeping the weight more evenly distributed between both feet and not raising the heel of the front foot too high off the ground increases stability. Raising the COG further stabilizes balance; however, rapid movements are more difficult to perform, and blocks are less effective. Finally, certain confounders such as physical condition, technical experience, and motor adaptation must be taken into consideration when applying the mentioned modifications to the various stances. For example, a lack of training routine with poor fitness and/or a low technical skill level must be excluded as a potential cause of inappropriate posture and stances. Moreover, the capability for motor adaption varies among athletes, and new techniques and stances need a certain technical skill level, i.e., repeated practice to ultimately be properly executed. Thus, caution should be applied before a suboptimal posture/stance or improper movement strategy is correlated to a pathologic spinopelvic alignment and sagittal imbalance. Particularly, the consultation of a physician or a health care professional is highly recommended when martial arts practitioners develop persistent pain of the spine and greater joints. A summary of the biomechanics of Goju-Ryu Karate-Do stances is given in Table 5. Further, the various stances including their challenges in aged or disabled practitioners with the respective modifications are given in Supplementary Figures S1–S4.

## 3. Conclusions

Goju-Ryu Karate-Do, a martial art form that combines hard and soft techniques, is strongly linked to the principles of balance, proper stances, and spinopelvic biomechanics. These elements are key to the successful execution of martial arts techniques, generating adequate stability and enough power. The optimal alignment of spinopelvic parameters is a prerequisite for maintaining an upright posture with minimal energy expenditure and a harmonious balance when performing various stances in Goju-Ryu Karate-Do. Both aging processes and degenerative diseases of the spine and greater joints lead to significant changes in the biomechanic parameters, affecting the ability to perform correct stances in Karate-Do and leading to decreased efficiency and balance. The body continuously compensates for these changes through mechanisms such as pelvic retroversion, knee flexion, and ankle extension to keep the body within the cone of economy. Proper knee flexion in karate stances is crucial for both performance and injury prevention. Therefore, training for aged or disabled practitioners should be adapted and focused on mastering the balance between stability and agility. This includes starting slowly, prioritizing stretching and warm-up exercises to increase the ROM of greater joints, emphasizing correct technique rather than brute force, allowing adequate rest, adapting stances to individual needs, and setting realistic goals. Thus, various modifications of the respective stances must be considered to prevent injury but also keeping aged individuals as active karatekas as long as possible. Particularly, preventing strains on the joints and muscles while still maintaining the effectiveness of the stance should be aimed for. Adapting the COG, while keeping balance and stability, facilitates the execution of stances with only a minor compromise on effectiveness. The relationship between martial arts and the biomechanics of spinopelvic alignment underscores the importance of understanding the principles of balance in the practice of martial arts. On the contrary, the concepts of balanced stances and movement strategies in martial arts might positively impact the outcome of rehabilitation of athletes. Conscious and holistic movement sequences with dynamic aspects (i.e., slow and fast techniques) force the human body to learn to perceive each single muscle group during a movement, leading to an improvement of both coordination and stability. Isolated training of single muscle groups to improve strength or monotonous endurance training like cycling, rowing, and/or jogging only improve raw strength and stamina; however, aspects like coordination, reflexes, and motor adaption are mostly neglected. The insights gained from this review contribute to the development of more effective training routines and adaptations for aged and disabled practitioners, ultimately enhancing the practice and mastery of Goju-Ryu Karate-Do with injury prevention.

## 4. Future Directions

Our article is the first to provide a synthesis of established biomechanic concepts of the spine and techniques and stances in Goju-Ryu Karate-Do. By integrating our expertise from both fields, a better understanding of balanced stances together with modifications for aged and disabled practitioners can be established. However, the impact of the suggested adaptations of stances should also be further investigated on a pathophysiological level, i.e., the improvement of the training experience for athletes with spine and/or hip disorders. Particularly, is it feasible to reduce pain and disability arising from the mentioned disorders by our suggested modifications of stances in martial arts and thus, maintain a positive training experience for aged and disabled athletes? Ideally, a prospective and controlled cohort trial focusing on practitioners with hip and/or knee osteoarthritis with and without the suggested modifications of stances might be able to prove the validity and applicability of biomechanic concepts from the spine to training experience in martial arts. In contrast to such investigations, a biomechanical analysis of the spinopelvic alignment and balance of the spine in Goju-Ryu Karate-Do athletes from various age groups could help in understanding a potential positive or negative effect of martial arts training on the spine itself. Are adult spine deformities which frequently origin from degenerative disease less frequent in martial arts practitioners than in the general population? Here, whole spine films using the EOS imaging system allow for the calculation of all of the necessary parameters of spinopelvic alignment and balance while these values could be readily compared with established normative values from healthy individuals and known cut-off values for adult spine deformity. Conclusively, our narrative review provides a first foundation to academically analyze the impact of Goju-Ryu Karate-Do practice on athletes and might also allow instructors, coaches, and physiotherapists to tailor the individual exercises with martial arts practitioners for a personalized approach.

## Figures and Tables

**Table 1:** Basic stances in Goju-Ryu Karate-Do.

Type of Stance	Description
Heiko-dachi (parallel stance)	Attention stance where the feet are shoulder-width apart, with the big toes and second toes facing forward. The inner edges of the feet are parallel, and the COG is equidistant from the two feet.
Sanchin-dachi (three-battle stance)	Challenging yet crucial stance in Goju-Ryu Karate-Do. The position of the heel of the front foot is on the same line as the toes of the back foot, with both feet slightly turned inward. The front foot is angled inward at approximately 20°.
Zenkutsu-dachi (front stance)	Long stance with one foot in front of the other, the front knee being bent and positioned above the center of the foot, and the back leg straight.
Shiko-dachi (sumo stance)	Wide and deep stance with feet being about two shoulder-widths apart, with the knees and toes pointing outwards at a 45° angle. The weight is evenly distributed on both legs, and the knees are deeply bent and pulled back (to the exterior) as far as possible.
Neko ashi-dachi (cat stance)	Stance with main weight (80%) on the rear foot while bending the knee, with the foot flat on the ground and the toes pointing outwards (30°). Almost no weight on the front leg (20%), which is about a shoulder-width away from the rear leg. The toes of the front foot point forward, the foot is flat on the ground, and the heel is raised.

**Table 2:** Compensatory mechanisms for sagittal imbalance.

Compensatory Mechanisms	Effect
Cervical hyperlordosis	Increased curvature in the neck to shift the head backward
Thoracic kyphosis reduction	Decreased thoracic kyphosis (mid-back curve) to restore balance
Lumbar hyperlordosis	Increased lumbar lordosis (lower back curve) to counterbalance
Pelvic retroversion	Pelvis tilts backward to align the spine
Knee flexion	Slight knee bending to shift the center of gravity
Ankle extension	Ankles extend to maintain stability

**Table 3:** Functional purposes of knee flexion and hip/ankle extension in karate stances.

Functional Purposes	Rationale
Stability and balance	Knee flexion lowers the COG and enhances stability. In Zenkutsu- or Shiko-dachi, bending the knees provides a solid base for maintaining balance during movements including the effective execution of blocks and strikes. Straight legs or a reduced ROM reduce the necessary stability and can compromise the ability to react appropriately.
Power generation	Explosive (ballistic) techniques rely on power and momentum generated via vigorous hip rotations stabilized by static legs (stances). Knee flexion stores energy in the muscles and tendons, which can be rapidly released during strikes. In Zenkutsu- or Kumite-dachi, deep knee flexion allows you to generate power for strong punches, kicks, and blocks.
Precision	Proper knee flexion ensures that your weight is distributed correctly. In Heiko-dachi, the front knee should be directly above the ankle, forming a stable triangle. Without adequate knee flexion, techniques may lack precision and power, and core balance might be swiftly lost.
Injury prevention	Some stances place significant stress on the great joints of the lower extremities. Controlled knee flexion helps to distribute forces evenly. Hyperextension or locking the knees should be avoided as this can strain ligaments and damage cartilage.

**Table 4:** Adaption of training for aged or disabled practitioners.

Adaptions	Description
Progressive start	Start training gradually. Avoid intense or sudden movements that may strain joints or muscles. Focus on mastering proper stances before progressing to more complex techniques.
Warm-up and stretching	Prioritize warm-up and stretching exercises. Gently stretch muscles to improve flexibility and prevent injuries. Warm-up prepares the body for training, especially for older joints and muscles. Increase the ROM of joints to increase compensatory mechanisms for core balance and for injury prevention.
Technique over power	Emphasize correct technique rather than employing brute force. Proper stances ensure stability and harmonious balance. Focus on precision to prevent strains.
Adequate rest	Perform repetitive rest periods during training. Allow time for recovery between exercises or repetitions. Listen to the body and avoid overexertion
Modified techniques	Adapt stances and techniques to individual needs. - Lower kicks: Perform kicks at a comfortable height. - Higher stances: Adjust stance height to reduce strains and maintain balance despite kyphosis of the spine and reduced ROM of joints. - Simplify movements to maintain safety and comfort.
Realistic goals	Set achievable goals based on age and fitness level with gradual progress. Continuity matters more than intensity.

**Table 5:** Biomechanics of Goju-Ryu Karate-Do stances.

Stance	Center of Gravity	Technical Demands	Limitations	Modifications
Heiko-dachi	Midline, anterior of second sacral vertebra	Low	None	None
Sanchin-dachi	Midline, slightly lowered and forward of front leg	Low	None	None
Zenkutsu-dachi	Lowered and forward over front leg	Moderate	Knee flexion, hip/ankle extension	Higher stance and decrease in length
Shiko-dachi	Maximally lowered and centered between feet	Moderate to high	Knee flexion, hip flexion and outward rotation	Higher stance, less outward rotation of hips
Neko ashi-dachi	Lowered and shifted over rear leg	High	Hip and ankle extension, knee flexion	Higher stance, less flexion of front feet

## Data Availability

No new data were created or analyzed in this study. Data sharing is not applicable to this article.

## Supplementary Materials

The following supporting information can be downloaded at: https://www.mdpi.com/article/10.3390/jfmk11010011/s1, Supplementary Figure S1: Heiko- and Sanchin-dachi. Front (Panels A and C) and side view (Panels B and D) of Heiko- and Sanchin-dachi. In Heiko-dachi the feet are shoulder-width apart, with the big toes and second toes facing forward. The inner edges of the feet are parallel, and the COG is equidistant from the two feet. In Sanchin-dachi, the position of the heel of the front foot is on the same line as the toes of the back foot, with both feet slightly turned inward. The front foot is angled inward at approximately 20°. Supplementary Figure S2: Zenkutsu-dachi. Long stance with one foot in front of the other, the front knee being bent and positioned above the center of the foot, and the back leg straight (Panel A: front view; Panel D: side view; red sphere: center of gravity [COG]). In aged or disabled practitioners, the range of motion (ROM) and strength in the lower extremities might be reduced with decreased lumbar lordosis. This results in an increased bending of the knee of the rear leg as well as a forward tilt of the upper body with a shift of the COG to the front (Panels B and E). Thus, the length of the stance can be shortened, and the front knee may be bent less deeply (Panels C and F). This lessens stress on the joints and muscles while still maintaining the effectiveness and balance of the stance. However, this raises the COG of the stance which makes it feel more stable, but it reduces the potential power which can be generated for attacks and blocks from this stance. Supplementary Figure S3: Shiko-dachi. Wide and deep stance with feet being about two shoulder-widths apart, with the knees and toes pointing outwards at a 45° angle. The weight is evenly distributed on both legs, and the knees are deeply bent and pulled back (to the exterior) as far as possible (Panel A: front view; Panel D: side view; red sphere: center of gravity [COG]). The stance requires a wide leg position with deep knee bends, which can be challenging for individuals with knee or hip issues. This might result in the knees as well as the upper body tilting forward. Consequently, the COG shifts to the front, accordingly (Panels B and E). Reducing the bending of the knees as well as the wideness of the stance reduces the stress on the greater joints. However, by raising the depth of the stance and thus the COG, balance is maintained but at the price of reduced stability (Panel C and F). Supplementary Figure S4: Neko ashi-dachi. Stance with main weight (80%) on the rear foot while bending the knee, with the foot flat on the ground and the toes pointing outwards (30°). Almost no weight on the front leg (20%), which is about a shoulder-width away from the rear leg. The toes of the front foot point forward, the foot is flat on the ground, and the heel is raised (Panel A: front view; Panel D: side view; red sphere: center of gravity [COG]). The stance demands balancing primarily on the posterior leg, which can be difficult for older individuals with reduced range of motion (ROM) of greater joints and balance issues. Particularly, the capability for ankle and hip extension limits the optimal performance of this stance which results in a tilt of the upper body to the front together with a shift of the COG to the front, accordingly (Panels B and E). Keeping the weight more evenly distributed between both feet and not raising the heel of the front foot too high off the ground increases stability. Additionally, raising the COG further stabilizes balance; however, rapid movements are more difficult to perform, and blocks are less effective (Panels C and F).

## Abbreviations

COG	Center of gravity
FBI	Full body integrated
LL	Lumbar lordosis
PI	Pelvic incidence
PT	Pelvic tilt
ROM	Range of motion
SGRKR	Swiss Goju-Ryu Karate-Do Renmei
SS	Sacral slope
SVA	Sagittal vertical axis
TK	Thoracic kyphosis
TPA	T1 pelvic angle
