# Supplementary figures and images for: Applying Principles of Biomechanics of the Spine to Martial Arts: A Review on Balance of Stances in Goju-Ryu Karate-Do
Source: J Funct Morphol Kinesiol. 2025 Dec 26;11(1):11. doi: 10.3390/jfmk11010011 (PMC12821703; doi:10.3390/jfmk11010011)

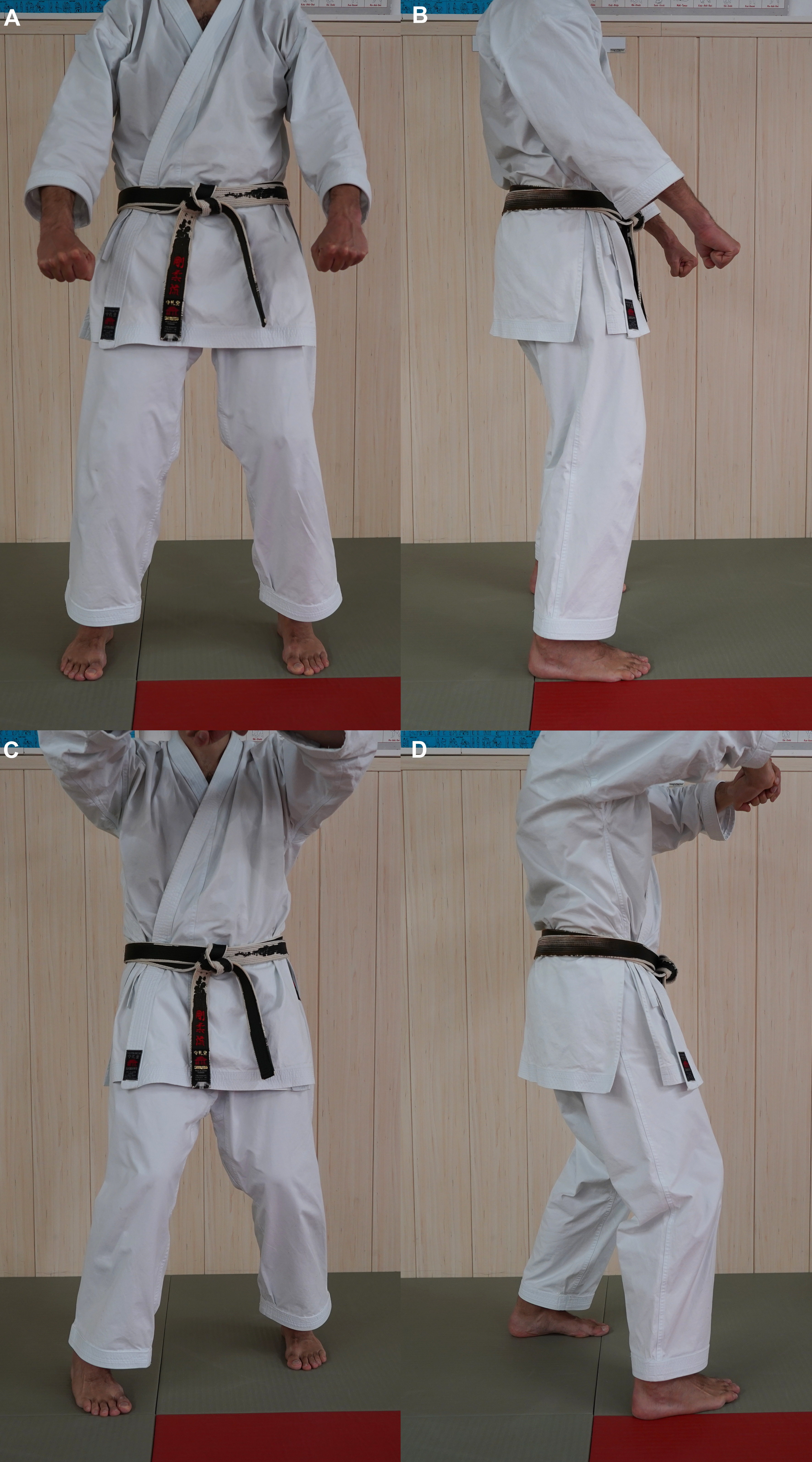

Supplement: Supplementary file 1 [file jfmk-11-00011-s001.zip › Supplementary Figure S1.tif]

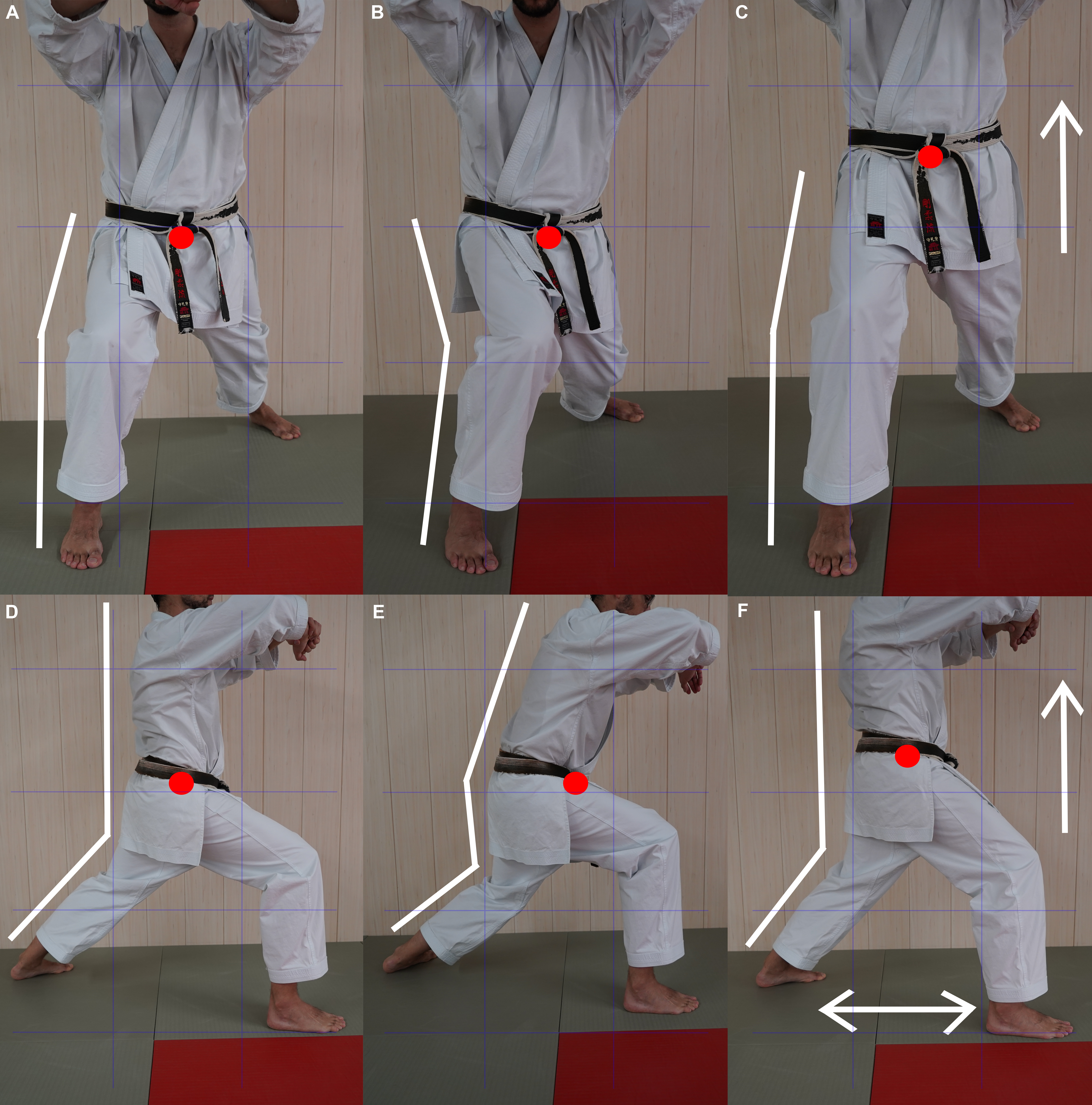

Supplement: Supplementary file 1 [file jfmk-11-00011-s001.zip › Supplementary Figure S2.tif]

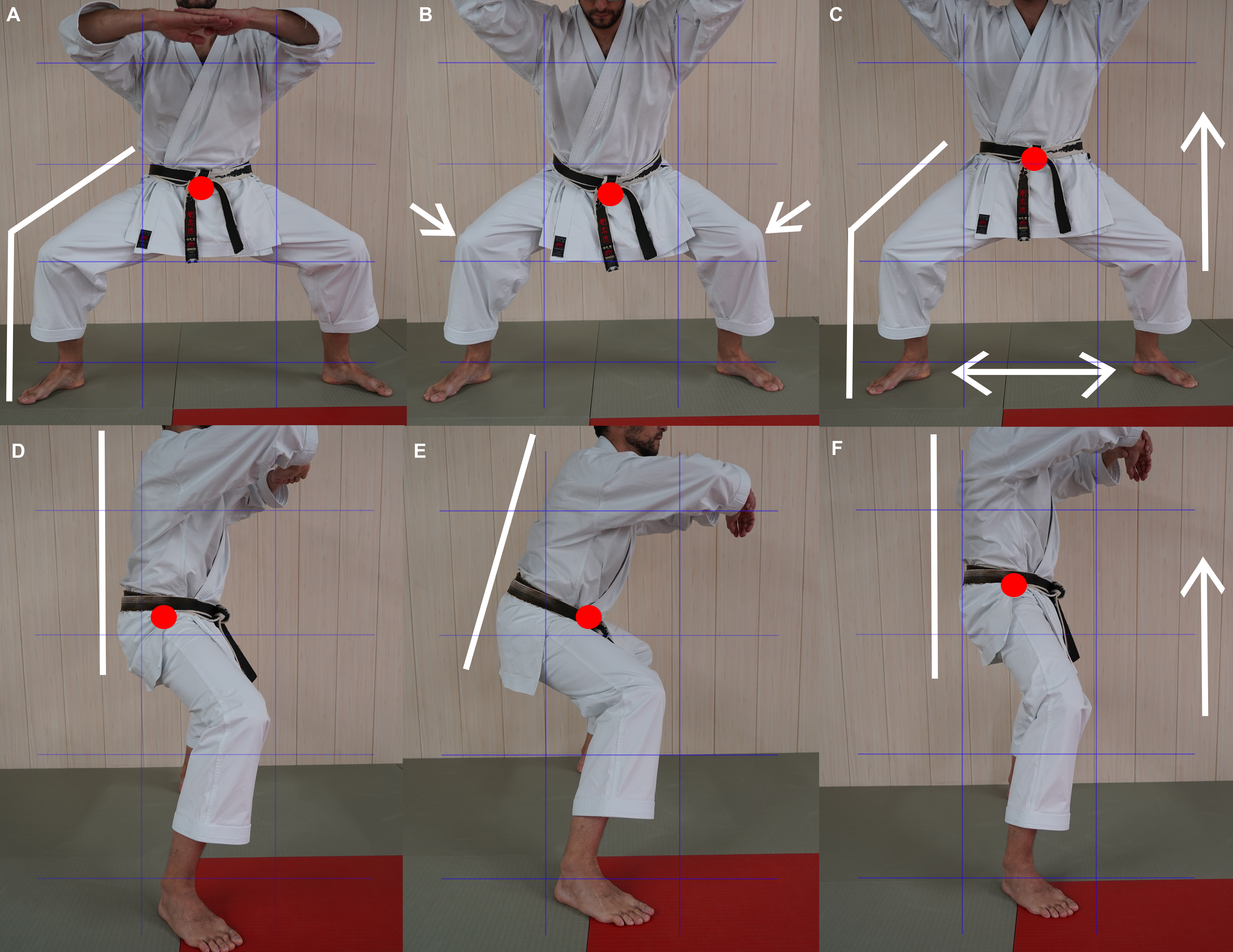

Supplement: Supplementary file 1 [file jfmk-11-00011-s001.zip › Supplementary Figure S3.tif]

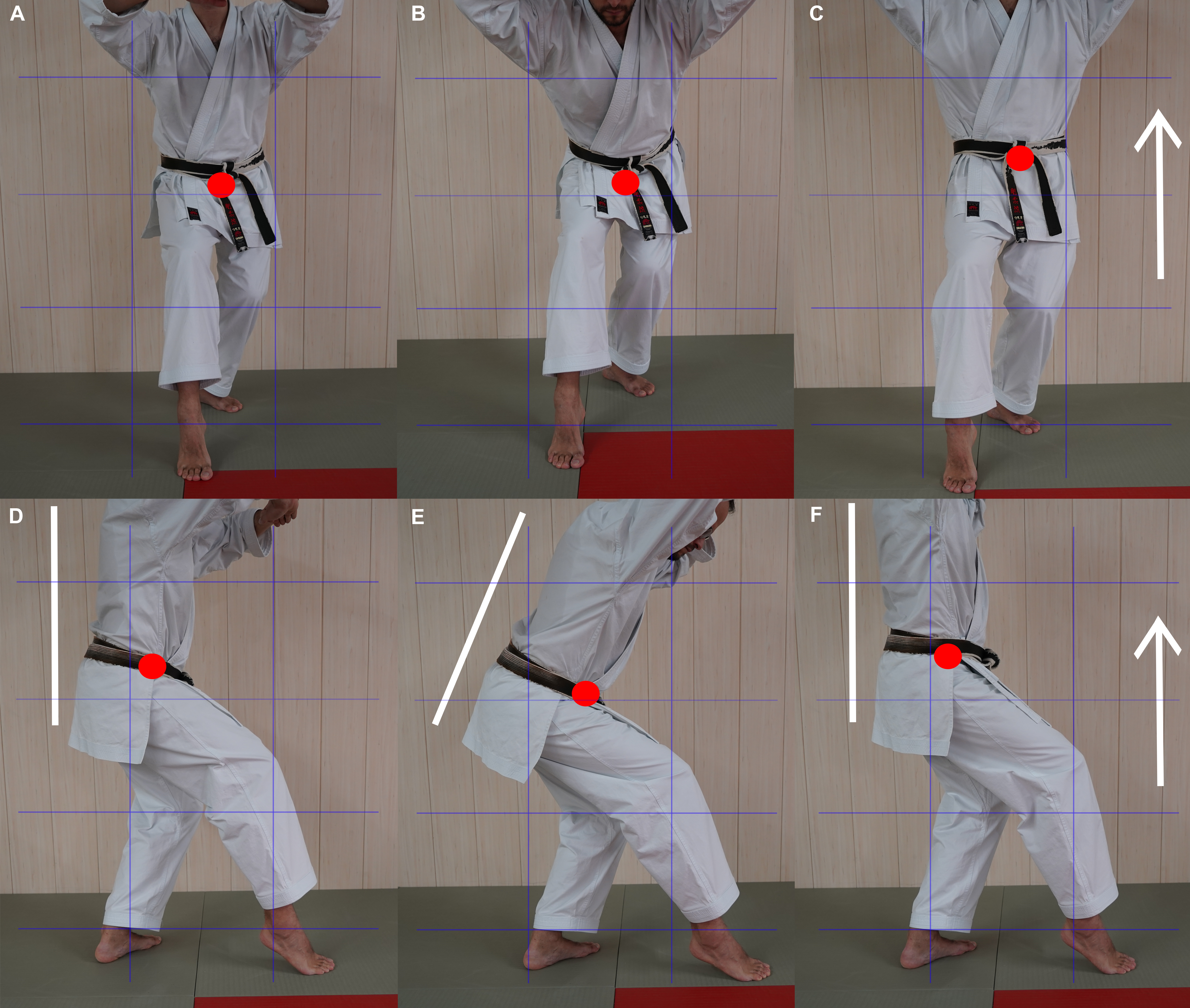

Supplement: Supplementary file 1 [file jfmk-11-00011-s001.zip › Supplementary Figure S4.tif]
